# Aminocyclopropane-1-carboxylic acid is a key regulator of guard mother cell terminal division in *Arabidopsis thaliana*

**DOI:** 10.1093/jxb/ery413

**Published:** 2018-11-21

**Authors:** Jiao Yin, Xiaoqian Zhang, Gensong Zhang, Yuanyuan Wen, Gang Liang, Xiaolan Chen

**Affiliations:** 1 School of Life Sciences, Yunnan University, Kunming, Yunnan, China; 2 CAS Key Laboratory of Tropical Plant Resources and Sustainable Use, Xishuangbanna Tropical Botanical Garden, Chinese Academy of Sciences, Kunming, Yunnan, China

**Keywords:** 1-Aminocyclopropane-1-carboxylic acid (ACC), ethylene, guard mother cell, stomata, symmetric division

## Abstract

Stomata have a critical function in the exchange of gases and water vapor between plants and their environment. Stomatal development is under the rigorous control of many regulators. The last step of development is the terminal division of guard mother cells (GMC) into two guard cells (GC). It is still unclear how the symmetric division of GMCs is regulated. Here, we show that the ethylene precursor aminocyclopropane-1-carboxylic acid (ACC) is required for the symmetric division of GMCs into GCs in Arabidopsis. Exogenous application of the ACC biosynthesis inhibitor aminoethoxyvinylglycine (AVG) induced the formation of single guard cells (SGCs). Correspondingly, an *acs* octuple-mutant with extremely low endogenous ACC also developed SGCs, and exogenous ACC dramatically decreased the number of SGCs in this mutant whereas exogenous ethephon (which is gradually converted into ethylene) had no effect. Furthermore, neither blocking of endogenous ethylene synthesis nor disruption of ethylene signaling transduction could induce the production of SGCs. Further investigation indicated that ACC promoted the division of GMCs in *fama-1* and *flp-1myb88* mutants whereas AVG inhibited it. Moreover, ACC positively regulated the expression of *CDKB1;1* and *CYCA2;3* in the *fama-1* and *flp-1myb88* mutants. The SGC number was not affected by ACC or AVG in *cdkb1;11;2* and *cyca2;234* mutants. Taken together, the results demonstrate that ACC itself, but not ethylene, positively modulates the symmetric division of GMCs in a manner that is dependent on CDKB1s and CYCA2s.

## Teaser

The ethylene precursor aminocyclopropane-1-carboxylic acid (ACC) positively regulates the symmetric division of stomatal guard mother cells in a manner that is dependent on CDKB1s and CYCA2s.

## Introduction

Stomata play a key role in controlling gas exchange between plants and the atmosphere. A stoma forms after at least one asymmetric division as well as a single symmetric division. Each asymmetric division produces one smaller cell (meristemoid) and one larger cell. The larger cell further divides unequally to extend the stem cell lineage. Meristemoids acquire a guard mother cell (GMC) fate after several rounds of asymmetric divisions. Stomatal development ends after the terminal division of a GMC, each of whose daughter cells terminally differentiates into a guard cell (Bergmann and Sack, 2007; Pillitteri and Torii, 2012). Guard cells exit the cell cycle at the G2-to-M stage. In Arabidopsis, A-type cyclins and B-type cyclin-dependent kinases function synergistically in the G2-to-M transition of GMCs (Boudolf *et al.*, 2004*a*, 2004*b*; Vanneste *et al.*, 2011). Transgenic Arabidopsis plants overexpressing a dominant negative allele of *CDKB1;1* (*CDKB1;1.N161*) have a block in GMC division and form single guard cells (SGCs). SGCs are also found on the epidermis of *cdkb1;11;2* and *cyca2;234* mutants. Although no phenotype is observed in the *cdkb1;1* single-mutant, the quadruple-mutant *cdkb1;1cyca2;234* displays more SGCs than the triple-mutant *cyca2;234*. CDKA;1 activity is also required for GMC division as evidenced by the fact that arrested GMC division is also found in the *cdka;1* null mutant (Weimer *et al.*, 2012). Expression of *CDKA;1* in the stomatal lineage driven by the *TOO MANY MOUTHS* (*TMM*) promoter can partially rescue *cdkb1;11;2* stomata defects, suggesting functional redundancy between CDKA;1 and CDKB1s. *CYCD3;2* and *CDKA;1* under the control of the *FAMA* (*bHLH097*) promoter can stimulate extra symmetric GC subdivisions, and their synergistic effect on promoting GC subdivision requires functional RETINOBLASTOMA RELATED (RBR) (Borghi *et al.*, 2010; Yang *et al.*, 2014).

FAMA and FOUR LIPS (FLP)/MYB88 are two classes of regulators for the symmetric division of GMCs. FAMA restricts the GMCs to a single division. Loss-of-function of *FAMA* causes cell clusters without GC identity. FAMA negatively regulates the expression of *CDKB1;1* and *CYCA2;3* via direct promoter binding (Hachez *et al.*, 2011; Lee *et al.*, 2014). Loss-of-function of *FLP* and *MYB88* also induces clusters of four or more guard cells (Lai *et al*, 2005). FLP/MYB88 restricts the symmetric division of GMCs to one event only by directly targeting the core cell cycle genes *CDKB1;1*, *CYCA2;3* and *CDKA;1* (Xie *et al.*, 2010; Vanneste *et al.*, 2011;Yang *et al.*, 2014). RBR is a negative regulator of cell cycle gene expression and cell proliferation (Inzé and De Veylder, 2006; Lee *et al.*, 2014). Both FAMA and FLP/MYB88 interact with RBR to form a complex to bind the upstream regulatory sequences of *CDKB1;1* (Lee *et al.*, 2014; Matos *et al.*, 2014).

Phytohormones play important roles in the growth and development of plants, and several regulate stomatal development, including brassinosteroids (BRs), abscisic acid (ABA), auxin, gibberellins (GAs), and jasmonates (JAs), as well as ethylene (Kieber *et al.*, 1993; Saibo *et al.*, 2003; Gudesblat *et al.*, 2012; Kim *et al.*, 2012; Khan *et al.*, 2013; Tanaka *et al.*, 2013; Balcerowicz *et al.*, 2014; Le *et al.*, 2014; Zhang *et al.*, 2014; Han *et al.*, 2018*b*).

The synthesis of ethylene starts from the conversion of methionine into S-adenosylmethionie, which is then converted by the enzyme 1-aminocyclopropane-1-carboxylate synthase (ACS) into methylthioadenosine (MTA) and aminocyclopropane-1-carboxylic acid (ACC). Ethylene is synthesized through the oxidation of ACC by ACC oxidase (ACO) (Dong *et al.*, 1992). Among the 12 genes (annotated as *ACS1*–*12*) in the Arabidopsis genome, *ACS3* is a pseudogene, and *ACS10* and *ACS12* encode aminotransferases (Yamagami *et al.*, 2003). Nine authentic *ACS* genes (*ACS1*, *ACS2*, *ACS4*–*9*, and *ACS11*) have been identified in the Arabidopsis genome (Tsuchisaka *et al.*, 2009). Each of these nine genes can contribute to ethylene production. The elimination of all of them results in embryonic lethality, but an *acs* octuple-mutant can survive (Tsuchisaka *et al.*, 2009).

In Arabidopsis, exogenous ACC induces a high density and different spacing of stomata on the epidermis of the cotyledon whilst the inhibition of ACC synthesis by aminovihylglycine (AVG) has the opposite effect (Serna and Fenoll, 1996). In addition, ACC treatment results in more stomata on the hypocotyls of Arabidopsis grown on low-nutrition medium (Saibo *et al.*, 2003). However, it is unclear whether ACC is related with the division processes of GMCs. Here, we show that ACC is required for the symmetric division of GMCs in Arabidopsis. Blocking ACC synthesis genetically or chemically resulted in the inhibition of GMC division and the formation of SGCs on the epidermis of cotyledons, leaves, and hypocotyls. Further analysis indicated that ACC-mediated GMC division is independent of ethylene and its signaling components, suggesting an independent role of ACC in the symmetric division in Arabidopsis. The positive role of ACC in the symmetric division of GMCs can be antagonized by FAMA and FLP/MYB88. Moreover, ACC functions in the division of GMCs in a manner that is dependent on CDKB1s and CYCA2s.

## Materials and methods

### Plant materials and growth conditions

*Arabidopsis thaliana* plants were grown on half-strength Murashige and Skoog (MS) medium at 22 °C under a 16/8 h light/dark photoperiod. Transgenic plants with GC-specific E1728, *pCDKB1;1::GFP*, or *pCYCA2;3::GFP* were obtained from Professor Fred Sack (University of British Columbia). The *acs* octuple-mutant harboring *E1728* was generated by genetic crossing using standard techniques, and the mutant seedlings were confirmed by PCR-based genotyping and YFP (Tsuchisaka *et al.*, 2009). Col-0 was used as the wild-type. Plants were sampled at 14 d after germination. In an additional experiment, plants were grown on low-nutrition medium (Smalle *et al.*, 1997) in the dark for 21 d to produce etiolated seedlings.

### Chemical treatments

Stock solutions were prepared by dissolving the following in DMSO: ACC at 10 mM; ethephon at 10 mM; AVG at 25 mM; α-aminoisobutyric acid (AIB) at 10 mM; and Co^2+^ at 25 mM (all chemicals obtained from Sigma). All the stock solutions were stored at –20 °C, and diluted with sterilized medium to the final experimental concentration: 10 μM for ACC, 10 μM for ethephon, 25 μM for AVG, 10 μM for AIB, and 25 μM for Co^2+^. Seeds were surface-sterilized and germinated on the treated medium or on control medium. Plants were then grown at 22 °C under a 16/8 h light/dark photoperiod.

### Microscopy

Images of stomata were obtained from samples stored in Hoyer’s Solution and visualized using differential interference contrast microscopy on a Nikon D-ECLIPSE C1 microscope. The cotyledons were observed at 14 d after germination. Samples were taken and placed in 70% ethanol, cleared overnight at room temperature, and then stored in Hoyer’s Solution. To visualize cell walls, freshly dissected leaves were stained for 3 min in an aqueous solution containing 2 mg ml^−1^ propidium iodide (Sigma-Aldrich). The leaves were then rinsed and mounted in distilled water. The abaxial side of leaves was viewed with a 3100 oil objective (Nikon Plan Fluor NA 1.3) using a Nikon PCM 2000 confocal laser-scanning microscope equipped with 488 nm argon and 543 nm helium–neon lasers. A Nikon D-ECLIPSE C1 laser confocal scanning microscope was used for green fluorescent protein (GFP) fluorescence images and propidium iodide staining.

### DAPI staining and measurement of DNA content

For analysis of nuclei in GCs, cotyledons were dissected and fixed in 70% ethanol for 3 h, incubated in DAPI staining solution for at least 30 min, and excited by UV fluorescence. Relative DNA levels were measured following the protocol described previously by Yang *et al.* (2014).

### Stomatal counts

Samples were fixed in Hoyer’s Solution and visualized using an Olympus BX51 microscope with a DP73 CCD camera. To determine numbers of SGCs, 50 cotyledons from 25 plants were counted for each genotype.

### RNA extraction and qRT-PCR

Total RNA was extracted from 4-d-old seedlings using Trizol reagent (Invitrogen). First-strand cDNA was synthesized from 1.5 μg DNase-treated RNA in a 20-μl reaction volumes using M-MuLV reverse transcriptase (Fermentas, EU) with an oligo(dT)18 primer. qRT-PCR was performed using 2× SYBR Green I Master Mix on a Roche Light Cycler 480 real-time PCR machine, according to the manufacturer’s instructions. At least three biological replicates for each sample were used for qRT-PCR analysis and at least three technical replicates were analysed for each biological replicate. The *KAT1* gene was used as an internal control (Nakamura *et al.*, 1995; Lee *et al.*, 2014). The primers used in qRT-PCR are listed in Supplementary Table S1 at *JXB* online.

### Accession numbers

The Arabidopsis Genome Initiative numbers for the genes considered in this article are as follows: *ACS1*, AT2G43750; *ACS2*, AT1G01480; *ACS3*, AT5G28360; *ACS4*, AT2G22810; *ACS5*, AT5G65800; *ACS6*, AT4G11280; *ACS7*, AT4G26200; *ACS8*, AT4G37770; *ACS9*, AT3G49700; *ASC10*, AT1G62960; *ACS11*, AT4G08040; *ETO1*, *AT3G51770*; *FAMA*, AT3G24140; *FLP*, AT1G14350; *MYB88*, AT2G02820; *CDKB1;1*, AT3G54180; *CDKB1;2*, AT2G38620; *CYCA2;2*, AT5G11300; *CYCA2;3*, AT1G15570; *CYCA2;4*, AT1G80370; and *KAT1*, AT5G46240.

## Results

### AVG treatment results in the disruption of GMC terminal division

**Fig. 1.**
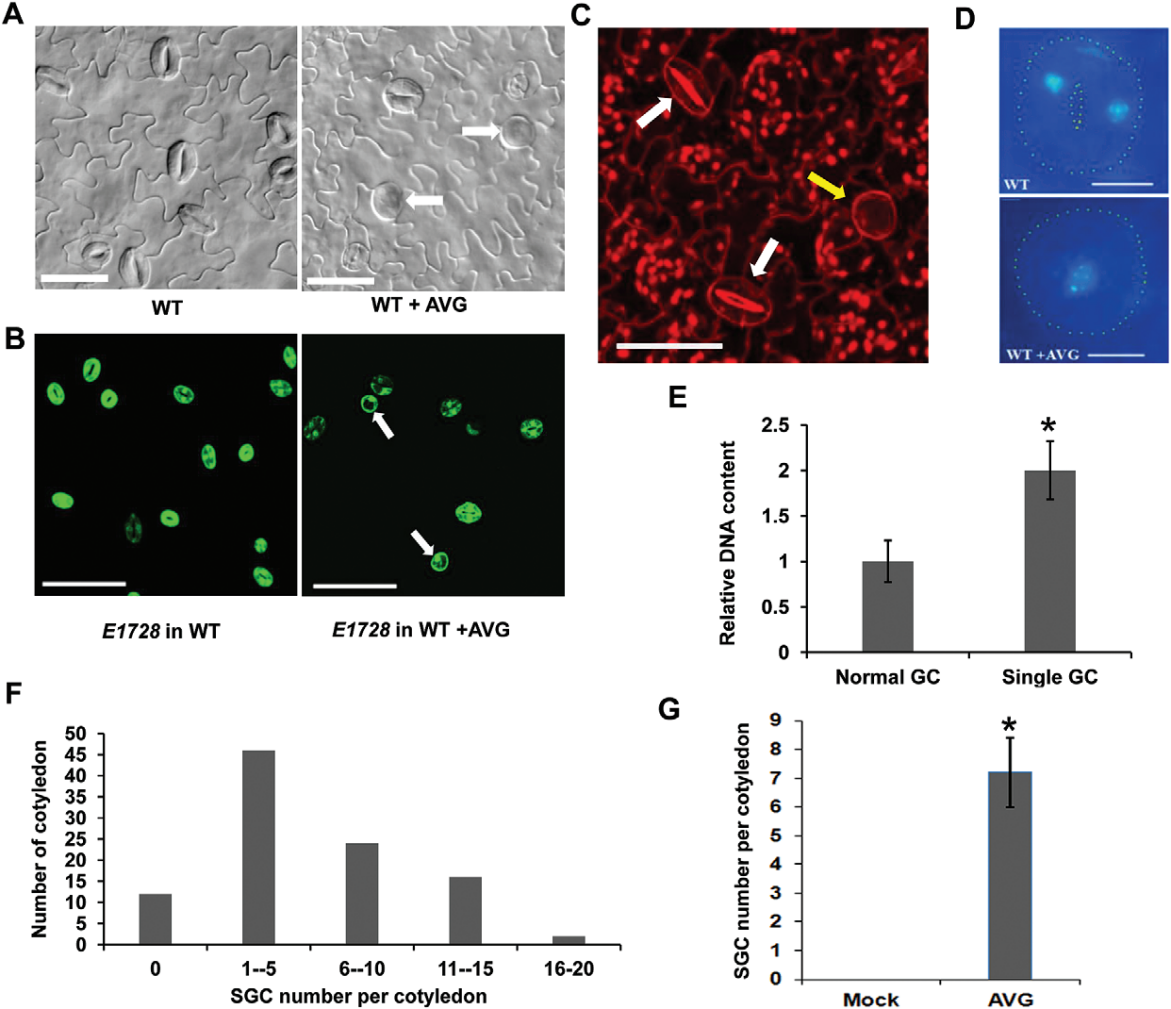
Treatment with aminoethoxyvinylglycine (AVG) results in the formation of single guard cells (SGCs). (A) Abaxial epidermis of cotyledons in the wild-type (WT) with or without AVG treatment. Arrows indicate SGCs. Scale bars are 20 μm. (B) *E1728* expression in the abaxial epidermis of cotyledons in the wild-type before and after AVG treatment. Arrow indicates SGCs. Scale bars are 100 μm. (C) Abaxial epidermis of a cotyledon in the wild-type with AVG treatment. The confocal *z*-projection was visualized using propidium iodide staining with a confocal laser-scanning microscope. The yellow arrow indicates a SGC without a central cell wall. White arrows indicate normal stomata with two GCs and a thickened central cell wall. The scale bar is 50 μm. (D) GCs and SGCs with DAPI fluorescence. Scale bars are 5 μm. (E) Quantitative analysis of DAPI fluorescence intensity in GCs and SGCs. The data are means (±SD) (*n*=10) and the significant difference between SGCs and GCs was determined using Student’s *t*-test: *P<0.01. (F) Frequency of cotyledons with different numbers of SGCs on the abaxial epidermis of AVG-treated wild-type plants. A total of 50 cotyledons from 25 seedlings were examined. (G) Mean number of SGCs per cotyledon in the wild-type with or without (Mock) AVG treatment. The significant difference between the means was determined using Student’s *t*-test: *P<0.01. A total of 50 cotyledons from 25 seedlings were examined.

It is known that ACC positively affects stomatal density (Serna and Fenoll, 1996; Saibo *et al.*, 2003); however, it is unknown whether it affects the division processes. Seedlings were therefore grown for 14 d on media with or without supplementary AVG, an ACC biosynthesis inhibitor. The seedlings grown with AVG developed some stomatal cells without a central pore (Fig. 1A, Supplementary Fig. S1), which are very similar to typical single guard cells (SGCs). To confirm whether these stomatal cells had the GC fate, wild-type seedlings harboring the GC fate marker *E1728er::YFP* (*E1728*) were treated with AVG, which could be seen to be expressed in the stomatal cells (Fig. 1B). We used propidium iodide to stain plant cell walls, which indicated that these abnormal stomatal cells had no internal cell wall (Fig. 1C). In addition, we used DAPI to examine the number and size of the nuclei, and found that they had only one nucleus (Fig. 1D) with nearly double the DNA content of normal GCs, as deduced from the fluorescent intensity of the DAPI-stained nuclei (Fig. 1E). All these results supported the identity of the cells as SGCs. To quantify the frequency of SGCs induced by AVG, we counted the numbers of SGCs and normal stomata on the abaxial epidermis of the cotyledons. No SGCs were found on the epidermis with stomata in any of the control seedlings. After AVG treatment, the number of SGCs varied from 0 to 16 per cotyledon (Fig. 1F) with an average number of 7.2 (Fig. 1G).

### Loss-of-function of *ACS* genes causes production of SGCs

**Fig. 2.**
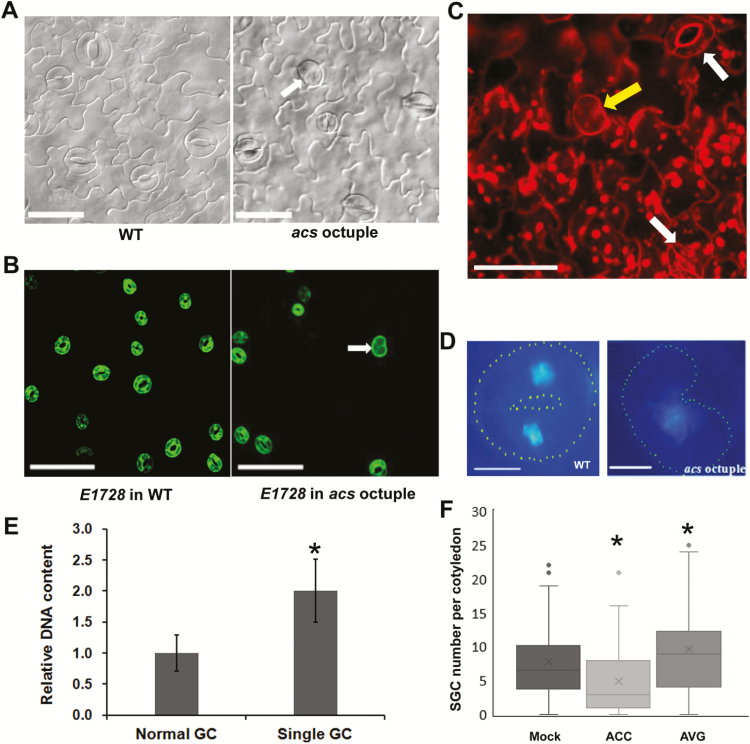
Defect of guard mother cell (GMC) division in the Arabidopsis *asc* octuple-mutant. (A) Abaxial epidermis of cotyledons of the wild-type (WT) and *acs* octuple-mutant. The arrow indicates a single guard cell (SGC). Scale bars are 20 μm (B) *E1728* expression in the abaxial epidermis of cotyledons of the wild-type and *acs* octuple-mutant. The arrow indicates a SGC. Scale bars are 100 μm. (C) Abaxial epidermis of a cotyledon of the *acs* octuple-mutant. The confocal *z*-projection image was visualized using propidium iodide staining with a confocal laser-scanning microscope. The yellow arrow indicates a SGC without a central cell wall. White arrows indicate normal stomata with two GCs and a thickened central cell wall. Scale bar is 50 μm. (D) GCs and SGCs imaged with DAPI fluorescence. Sacle bars are 5 μm. (E) Quantitative analysis of DAPI fluorescence intensity in GCs and SGCs. The data are means (±SD) (*n*=10) and the significant difference between SGCs and GCs was determined using Student’s *t*-test: *P<0.01. (F) Number of SGCs per cotyledon in the *acs* octuple-mutant in control (Mock) plants and plants treated with aminocyclopropane-1-carboxylic acid (ACC) and aminoethoxyvinylglycine (AVG). The data are means (±SD) and significant differences compared with the Mock were determined using Student’s *t*-test: *P<0.01. A total of 50 cotyledons from 25 seedlings were analysed.

To further investigate the functions of ACC, an *acs* octuple-mutant (CS16651: *acs2-1/4-1/5-2/6-1/7-1/9-1 amiRacs8 acs11*) with rather low endogenous ACC was used for analysis. As expected, putative SGCs were found on the epidermis of cotyledons, true leaves, and hypocotyls in this mutant (Fig. 2A, Supplementary Fig. S2). These abnormal stomatal cells were further identified as SGCs according to their DNA content, GC fate, and cell walls (Fig. 2B–E). The number of SGCs on the abaxial epidermis of cotyledons ranged from 0 to 25 in the *acs* octuple mutant (Fig. 2F).

Given the low level of ACC in the *acs* octuple-mutant, we then sought to determine whether addition of ACC could rescue the defect of GMC division. As expected, exogenous ACC treatment effectively reduced the number of SGCs on the abaxial epidermis of the cotyledons (Fig. 2F). In contrast, AVG treatment increased the number of SGCs. These data suggest that ACC is required for the division of GMCs into GCs.

### Blocking ethylene synthesis or signaling fails to cause SGC formation

**Fig. 3.**
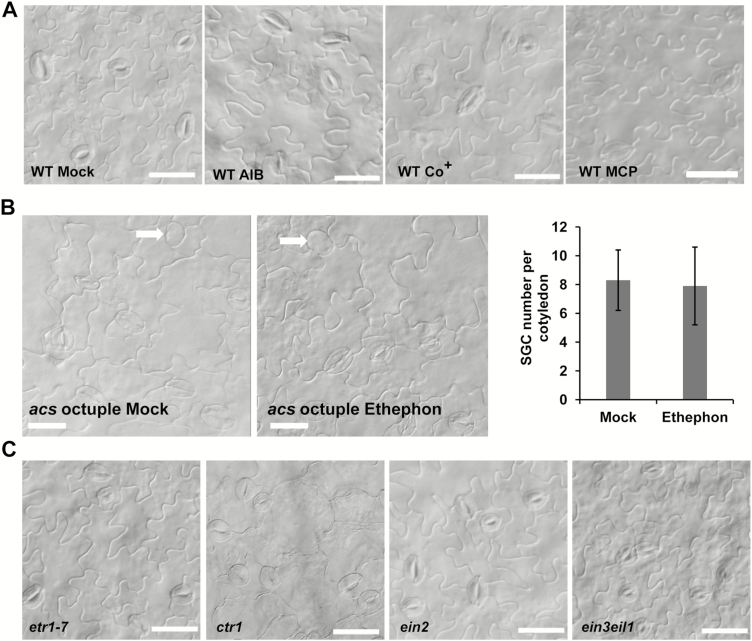
Blocking ethylene synthesis and signaling has no effect on guard mother cell division in Arabidopsis wild-type (WT) and *acs* octuple-mutant plants. (A) Abaxial epidermis of the wild-type after treatment with α-aminoisobutyric acid (AIB), Co^2+^, or 1-methycyclopropene (1-MCP) compared with untreated controls (Mock). Scale bars are 20 μm. (B) Numbers of single guard cells (SGCs) of abaxial epidermis of the *acs* octuple-mutant after treatment with ethephon. Arrow indicates SGCs. Scale bars are 20 μm. Data are are means (±SD). (C) Abaxial epidermis of the ethylene signaling-associated mutants *etr1-7*, *ctr1*, *ein2*, and *ein3eil1*. Scale bars are 20 μm.

Having confirmed that ACC was required for the terminal division of GMCs, we investigated whether ethylene also played a role similar to ACC by using ethylene-synthesis inhibitors to block its production. ACO catalyses the oxidation of ACC into ethylene and this reaction can be inhibited by α-aminoisobutyric acid (AIB). Low concentrations of AIB can efficiently inhibit the ACC binding protein while millimolar concentrations can block ACC oxidase (Xu *et al.*, 2008). To our surprise, AIB treatment failed to result in SGC production in the wild-type seedlings, even under a high concentration of 10 mM (Fig. 3A). Similar to AIB, Co^2+^ is also an inhibitor of ethylene synthesis, and no SGCs were found in the wild-type seedlings treated with Co^2+^ (Fig. 3A). These data suggested that inhibition of ethylene synthesis through ACC oxidation has no contribution to the formation of SGCs.

Given that ACC could partially rescue the stomatal division defect in the *acs* octuple-mutant, we examined whether ethylene could also play a similar role. Ethephon (a widely used chemical replacement for ethylene) was used to treat seedlings. As expected, ethephon treatment caused the typical triple response that is inductive of high levels of ethylene (data not shown); however, the number of SGCs in the *acs* octuple-mutant was not significantly affected (Fig. 3B).

Next, we sought to determine whether ethylene signaling was required for the terminal division of GMCs. 1-methycyclopropene (1-MCP) is a highly specific and competitive inhibitor of ethylene binding to receptors (Hall *et al.*, 2000; Sisler and Serek, 2006). As shown in Fig. 3A, no SGCs were found on the epidermis of seedlings subjected to 1-MCP treatment. To further confirm that ethylene signaling was not necessary for the symmetric division of GMCs, we checked the shape of stomata in several ethylene-signaling mutants. ETR1 (ETHYLENE RECEPTOR 1) is an ethylene receptor, CTR1 is a negative regulator of the ethylene response pathway, and EIN2 (ETHYLENE INSENSITIVE 2) and EIN3 (ETHYLENE INSENSITIVE 3)/EIL1 (ETHYLENE-INSENSITIVE3-LIKE 1) mediate the ethylene signaling transduction (Chang *et al.*, 1993; Kieber *et al.*, 1993; Hua and Meyerowitz, 1998; Hua *et al.*, 1998; Sakai *et al.*, 1998). As expected, no SGCs were observed in the ethylene-signaling mutants *etr1-7*, *ctr1*, *ein2*, and *ein3eil1* (Fig. 3C). These data suggested that neither ethylene itself nor ethylene-signaling components affect the terminal division of GMCs.

### ACC fails to induce extra divisions of the GMCs in wild-type Arabidopsis

**Fig. 4.**
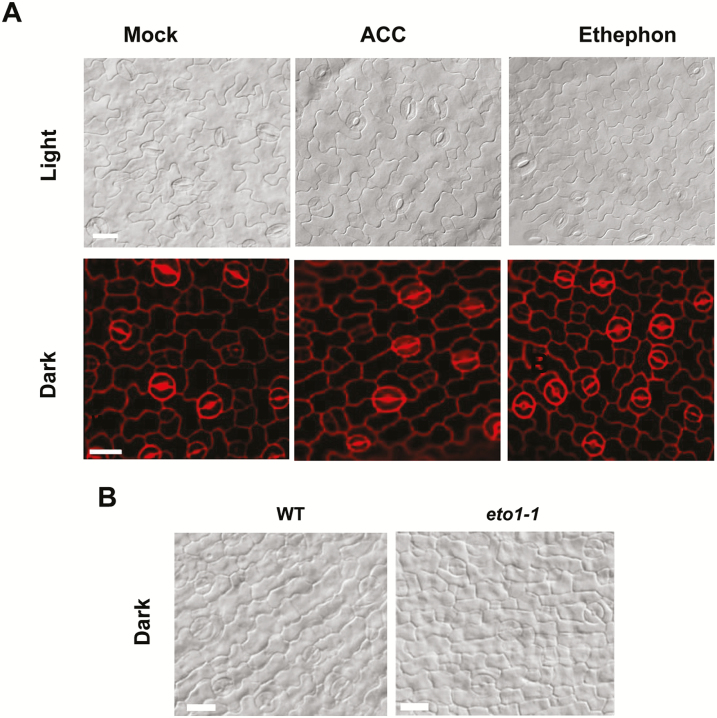
Elevation of aminocyclopropane-1-carboxylic acid (ACC) and ethylene fails to induce extra division of guard mother cells in wild-type (WT) Arabidopsis seedlings. (A) Abaxial epidermis of cotyledons. Seedlings were grown on media with ACC or ethephon under a 16/8 h photoperiod or under constant darkness. Scale bars are 20 μm. (B) Abaxial epidermis of cotyledons of the wild-type and the *eto1-1* mutant grown under constant darkness. Scale bars are 20 μm.

Having confirmed that ACC was required for the last symmetric division of GMCs, we examined whether elevated ACC would lead to extra divisions of GMCs. We treated wild-type seedlings with ACC, as well as with ethephon. No extra divisions of GMCs were found in either treatment for seedlings grown under a 16/8 h light/dark photoperiod (Fig. 4A, Supplementary Fig. S3).

A previous study found that exogenous ethylene treatment causes ectopic division of GMCs on the hypocotyl epidermis of etiolated cucumber seedlings grown on low-nutrient medium (Kazama *et al.*, 2004). Therefore, to exclude the possible effect of light, we examined the stomatal lineage cells of etiolated seedlings grown on low-nutrient medium with ACC or ethephon. We did not find extra divisions of GMCs (Fig. 4A, Supplementary Fig. S3). These results suggested that exogenous ACC has no effect on the terminal division of GMCs in Arabidopsis.

We then examined whether an increase of endogenous ACC or ethylene would cause ectopic division of GMCs by using, the ethylene over-accumulating mutant *eto1* (*ethylene overproducer1*), which has enhanced ACS activity and over-produces both ACC and ethylene (Guzmán and Ecker, 1990; Woeste *et al.*, 1999). We found that all the stomata on the epidermis of both the cotyledons and the hypocotyls consisted of two symmetric GCs without extra division (Fig. 4B; Supplementary Fig. S4). Taken together, these data suggested that a high level of ACC is not sufficient for the induction of extra symmetric divisions of the GMCs.

### ACC causes extra divisions of the GMCs in *fama-1* and *flp-1myb88* mutants

**Fig. 5.**
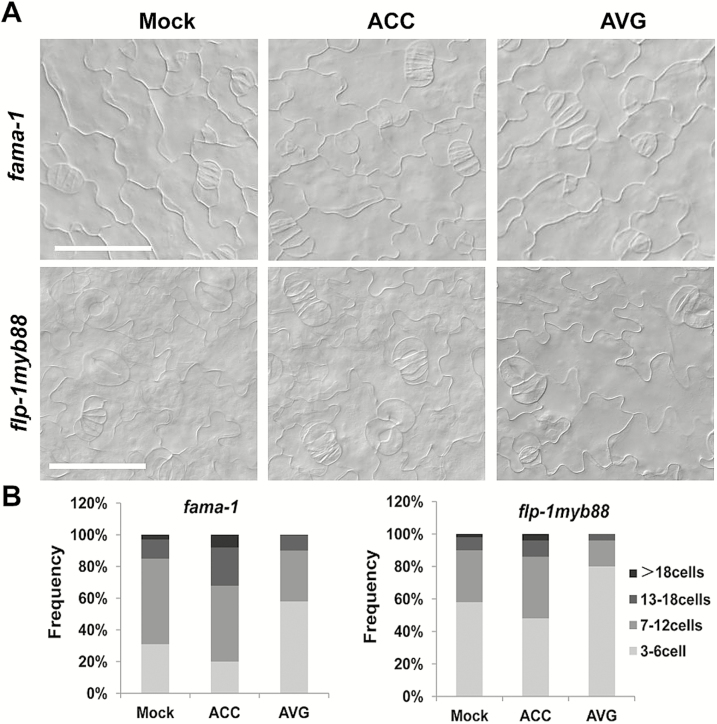
Aminocyclopropane-1-carboxylic acid (ACC) promotes symmetric division of guard mother cells in Arabidopsis *fama-1* and *flp-1myb88* mutants. (A) Abaxial epidermis of cotyledons of the mutants treated with ACC or aminoethoxyvinylglycine (AVG) compared with the control (Mock). Scale bars are 20 μm. (B) Frequency of cell clusters containing different numbers of cells on the abaxial epidermis of *fama-1* and *flp-1myb88* mutants treated with ACC or AVG.

Failure of ACC to induce more divisions of GMCs indicated that a rigid genetic mechanism existed to restrict these division. In the known genetic regulation network for stomatal development in Arabidopsis, FAMA and FLP/MYB88 are the key regulators of GMC division. FAMA is required to control the transition from GMCs to GCs and to halt division at the end of the stomatal lineage. The *fama-1* mutant lacks recognizable stomata and instead develops clusters of small, narrow epidermal cells where they would normally be located (Ohashi-Ito and Bergmann, 2006). We therefore examined whether ACC could promote the symmetric division of GMCs in the *fama-1* mutant. We found that the stomatal clusters of *fama-1* contained more cells after ACC treatment (Fig. 5A, B), suggesting that it promoted the division of GMCs. When AVG was applied, the stomatal clusters developed fewer cells, suggesting that it suppressed the division of GMCs. In addition to FAMA, FLP and MYB88 also restrict the symmetric division of GMCs (Lai *et al.*, 2005). To further investigate whether FLP and MYB88 are required for the role of ACC in the division of GMCs, the *flp-1myb88* mutant was used for analysis. Loss-of-function of *FLP/MYB88* induced excessive GMC divisions and produced many stomatal cell clusters (Fig. 5A, B). When the *flp-1myb88* mutant was treated with ACC, more cells occurred in the stomatal clusters, whilst in contrast AVG treatment inhibited the excessive divisions and decreased the number of cells in the clusters. When ethephon, AIB, and Co^2+^ were used to treat seedlings, the division of stomatal clusters was not affected in the *fama-1* and *flp-1myb88* mutants (Supplementary Fig. S5). The promotion of division of stomatal clusters in the *fama-1* and *flp-1myb88* mutants by ACC was not observed in the wild-type, implying that functional FAMA and FLP/MYB88 antagonize the positive role of ACC in controlling the division of GMCs.

### ACC promotes the expression of *CDKB1* and *CYCA2*

**Fig. 6.**
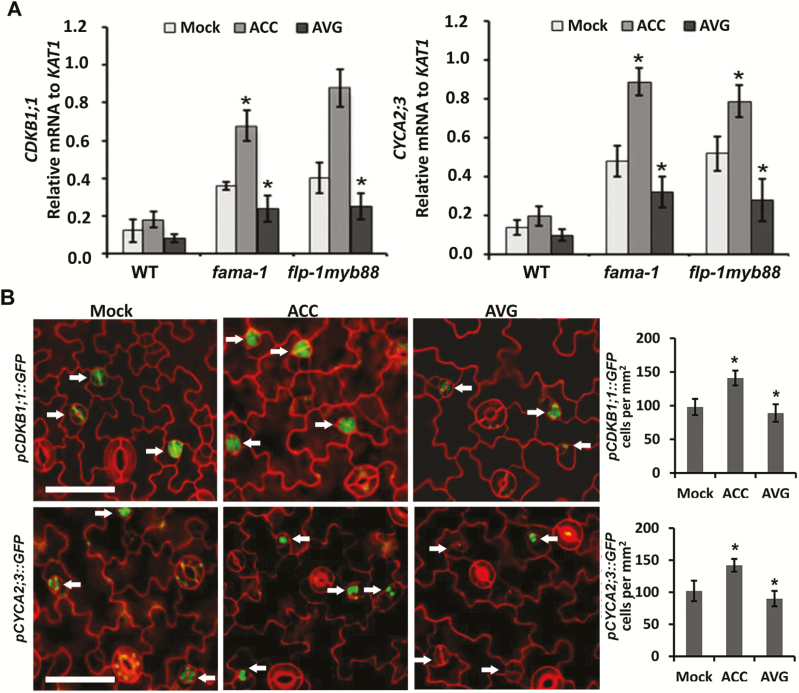
Aminocyclopropane-1-carboxylic acid (ACC) positively regulates expression of *CDKB1;1* and *CYCA2;3* in Arabidopsis. (A) Relative mRNA levels of *CDKB1;1* and *CYCA2;3* (normalized to *KAT1*) in the wild-type (WT) and *fama-1* and *flp-1myb88* mutants treated with ACC or aminoethoxyvinylglycine (AVG) compared with the control (Mock). Data are means (±SD) of three biological replicates. Significant differences compared with the corresponding Mock value were determined using Student’s *t*-test: *P<0.01. Three independent experiments were performed. (B) Expression of *pCDKB1;1::GFP* and *pCYCA2;3::GFP*. Representative images are shown. Arrows indicate newly formed GCs. The graphs show quantitative analyses of GFP-positive cells. Data are means (±SD) of three biological replicates. Significant differences compared with the Mock were determined using Student’s *t*-test: *P<0.01. A total of 50 cotyledons from 25 seedlings were analysed. Three independent experiments were performed.

It is well known that FAMA and FLP/MYB88 can restrict the division of stomatal lineage cells by negatively regulating the expression of *CDKB1;1* and *CYCA2;3* (Xie *et al.*, 2010; Hachez *et al.*, 2011; Vanneste *et al.*, 2011; Lee *et al.*, 2014). We hypothesized that ACC might stimulate the expression of *CDKB1;1* and *CYCA2;3* and thus promote the division of GMCs. To test this, we determined the expression of *CDKB1;1* and *CYCA2;3* in the wild-type, and *fama-1* and *flp-1myb88* mutants after ACC or AVG treatment (Fig. 6A). Under control conditions, the expression of *CDKB1;1* and *CYCA2;3* was significantly higher in *fama-1* and *flp-1myb88* than in the wild-type, agreeing with negative regulation by FAMA and FLP/MYB88. After ACC treatment, the expression of *CDKB1;1* showed no significant change in wild-type, but increased in *fama-1* and *flp-1myb88* (by 2.4- and 2.5-fold, respectively). A similar effect was observed for the expression of *CYCA2;3* after ACC treatment. In contrast, AVG inhibited the expression of *CDKB1;1* and *CYCA2;3* in the *fama-1* and *flp-1myb88* mutant plants. Correspondingly, we also detected GFP signals in *pCDKB1;1::GFP* and *pCYCA2;3::GFP* plants, with the signal increasing after ACC treatment and decreasing after AVG treatment (Fig. 6B). These data suggested that ACC stimulates the expression of *CDKB1;1* and *CYCA2;3*.

### ACC has no effect on the division of GMCs in *cdkb1;11;2* and *cyca2;234* mutants

**Fig. 7.**
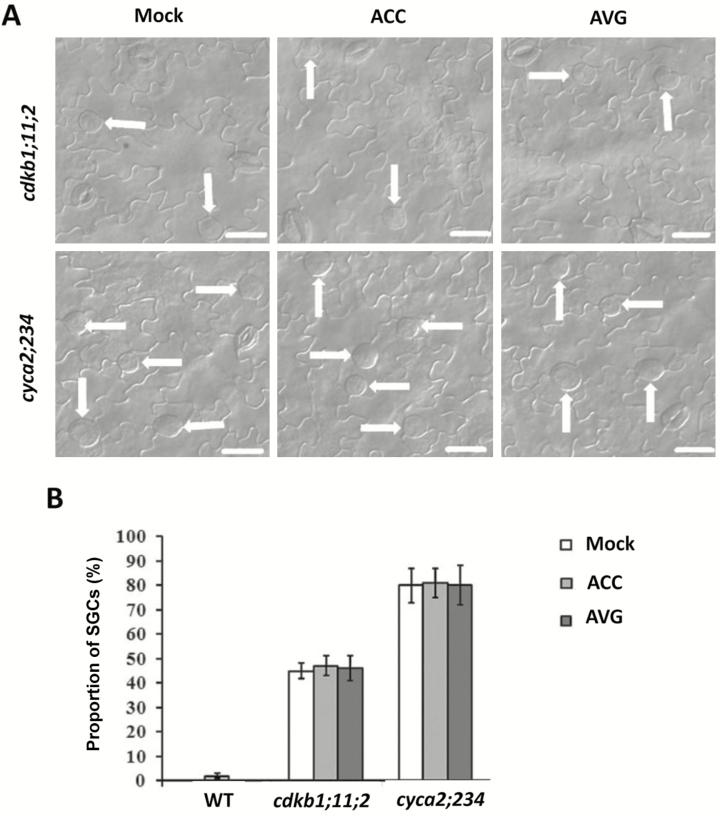
The *cdkb1;11;2* and *cyca2;234* mutants are insensitive to treatment with aminocyclopropane-1-carboxylic acid (ACC) and aminoethoxyvinylglycine (AVG). (A) Abaxial epidermis of cotyledons of the *cdkb1;11;2* and *cyca2;234* mutants treated with ACC or AVG compared with the control (Mock). The arrows indicate single guard cells (SGCs). Scale bars are 20 μm. (B) Proportion of SGCs in the *cdkb1;11;2* and *cyca2;234* mutants and the wild-type (WT) treated with either ACC or AVG. Data are means (±SD) (*n*=10).

Given the fact that *CDKB1;1* and *CYCA2;3* were induced by ACC treatment, we were interested to determine whether the function of ACC in promoting the division of GMCs was dependent on *CDKB1*s and *CYCA2*s. We therefore used the *cdkb1;11;2* and *cyca2;234* mutants for further analysis. Under normal growth conditions, SGCs accounted for about 45% of stomata on the epidermis of cotyledon in the *cdkb1;11;2* mutant (Fig. 7A, B) and we found that the number and proportion were not affected by ACC and AVG. On the abaxial epidermis of cotyledons of the *cyca2;234* mutant, about 80% of stomata were SGCs and again neither ACC nor AVG treatment changed the proportion (Fig. 7A, B). These data suggested that *CDKB1*s and *CYCA2*s are required for the function of ACC in promoting the division of GMCs.

## Discussion

### ACC, but not ethylene, is required for the terminal division of GMCs

Ethylene is a pivotal plant hormone that regulates multiple processes of plant growth and development. ACC is the direct precursor of ethylene production; however, it has also been identified as a primary regulator of plant growth and development (Vanderstraeten and Van Der Straeten, 2017). For example, in contrast to the viability of null mutations in key components of the ethylene-signaling pathways, such as the *ein2* and *ctr1* mutants (Kieber *et al.*, 1993; Roman *et al.*, 1995; Alonso *et al.*, 1999), the *acs* null mutant is lethal, strongly supporting the hypothesis that ACC, but not ethylene, is required for Arabidopsis viability (Tsuchisaka *et al.*, 2009). In addition, ACC is the primary regulator responsible for cell expansion mediated by the SALT OVERLY SENSITIVE 5 (SOS5)/FEI pathway in Arabidopsis roots. The cell expansion defect in the roots of *fei1fei2* is suppressed by the inhibition of ACC synthase, but not by the inhibition of ACC oxidase or by disruption of the ethylene response pathway, indicating that the FEI proteins regulate cell extension via an ACC-mediated signal (Xu *et al.*, 2008; Tsang *et al.*, 2011).

Although ethylene and ACC have been reported to regulate stomatal development, it is still unclear whether they affect the division of stomatal lineage cells. AVG is a direct inhibitor of the conversion of S-adenosylmethionie to ACC. When AVG was applied, SGCs formed on the epidermis of wild-type plants (Fig. 1). Correspondingly, the *acs* octuple-mutant in which ACC production is severely blocked also developed SGCs (Fig. 2). It is noteworthy that ACC, but not ethephon, reduced the number of SGCs in the *acs* octuple-mutant (Figs 2F, 3B) These data suggest that ACC is crucial for the normal division of GMCs. In contrast, the direct block of ethylene synthesis by AIB or Co^2+^ did not result in the formation of SGCs (Fig. 3A), implying that ethylene is not involved in the control of GMC division. In agreement with these results, the disruption of the binding of ethylene with its receptors by 1-MCP also failed to induce SGC formation (Fig. 3A). Similarly, no SGCs were found in the *etr1-7*, *ctr1*, *ein3*, and *ein3eil1* mutants, which are defective in ethylene perception or signaling (Fig. 3C). Taken together, these data suggest that ACC, but not ethylene, is required for the symmetric division of GMCs.

### Antagonistic roles between ACC and FAMA/FLP/MYB88 in the symmetric division of GMCs in Arabidopsis

The production of ACC and ethylene is greatly inhibited in all multiple *acs* mutants including the octuple mutant. Ethylene production is decreased by 92% and 86% in seedlings and mature plants, respectively, of the *acs* octuple-mutant (Tsuchisaka *et al.*, 2009). We only found SGCs in the *acs* octuple-mutant and not in other lower-multiple mutants, including a variety of double-, quadruple-, pentuple-, hexuple-, and even heptuple-mutants (Supplementary Fig. S6), which suggests that a rather low level of endogenous ACC is sufficient for the division of GMCs. We observed that the frequency of SGCs was quite low in the octuple-mutant plants (Fig. 2F), which means that a large portion of GMCs undergo normal symmetric division. It is likely that a complete absence of endogenous ACC would result in more SGCs; however, the exclusion of all the nine functional *ACS* genes causes embryo lethality (Tsuchisaka *et al.*, 2009). We also cannot exclude the possibility that other factor(s) might function in positively regulating the terminal division of GMCs in Arabidopsis.

Loss-of-function of *FAMA* or *FLP/MYB88* increased the division frequencies of GMCs in Arabidopsis, resulting in more cells in the clusters (Fig. 5). ACC treatment increased the division frequencies and the cell number of clusters in both the *fama-1* and *flp-1myb88* mutants. In contrast, ACC had no effect on the division of GMCs in the wild-type (Fig. 4). Therefore, FLP/MYB88 and FAMA antagonized the positive role of ACC in promoting the division of GMCs. The expression of *CDKB1;1* and *CYCA2;3* in GMCs was under the control of FAMA, FLP, and MYB88. FLP/MYB88 and FAMA directly bind to the promoter sequences of *CDKB1;1* and *CYCA2;3* and inhibit their expression in the newly formed GCs after the terminal division, limiting the division to a single event (Xie *et al.*, 2010; Vanneste *et al.*, 2011; Lee *et al.*, 2014). In contrast to the negative roles of FLP/MYB88 and FAMA, ACC positively regulated the expression of *CDKB1;1* and *CYCA2;3* (Fig. 6). It has previously been confirmed that CDKB1;1 and CYCA2;3 are needed for the GMCs to progress through the G2-to-M transition (Porceddu *et al.*, 2001; Boudolf *et al.*, 2004*a*; Vanneste *et al.*, 2011). After AVG treatment, the division of GMCs halted and caused the formation of SGCs (Fig. 1A, B), in agreement with the negative regulation of *CDKB1;1* and *CYCA2;3* by AVG (Fig. 6). ACC partially rescued the defect of GMC division in the *acs* octuple-mutant (Fig. 2F), but not that of the *cdkb1;11;2* and *cyca2;234* mutants (Fig. 7), which suggests that the promotion of GMC division by ACC depends on CDKB1;1 and CYCA2;3. *CDKB1;1* and *CYCA2;3* are specifically expressed in the stomatal lineage cells, with a maximum expression level during the symmetric division. We found that ACC enhanced the expression of *CDKB1;1* whereas AVG weakened its expression in the newly formed GCs (Fig. 6B). *CYCA2;3* displayed expression patterns similar to *CDKB1;1* after ACC and AVG treatments. In contrast, the expression of *CDKB1;1* and *CYCA2;3* did not change with or without treatment with ethephon, AIB, or Co^2+^ in either the wild-type or the ethylene signaling mutants *ein2* and *ein3eil1* (Supplementary Fig. S7).These data suggest that ACC bypasses the ethylene-signaling pathway to promote the division of GMCs via the activation of *CDKB1;1* and *CYCA2;3*. Further investigation is required to identify the unknown transcription factors that are responsible for the up-regulation of *CDKB1;1* and *CYCA2;3* by ACC.

In cucumber, extra divisions of GMCs have been observed on the hypocotyl epidermis after ethylene treatment (Kazama *et al.*, 2004). However, we found that this phenomenon occurred only in Arabidopsis without functional FAMA or FLP/MYB88 (Fig. 5). The different roles of ACC in Arabidopsis and cucumber may result from their different stomatal development processes. For example, in Arabidopsis the stomatal lineage precursor cells undergo at least one asymmetric division to produce a GMC, while in cucumber no asymmetric division is needed for the formation of GMCs (Kazama *et al.*, 2004). We hypothesize that this species-specific stomatal development processes may contribute to their different responses to ACC.

### A working model for ACC mediation of GMC division in Arabidopsis

**Fig. 8.**
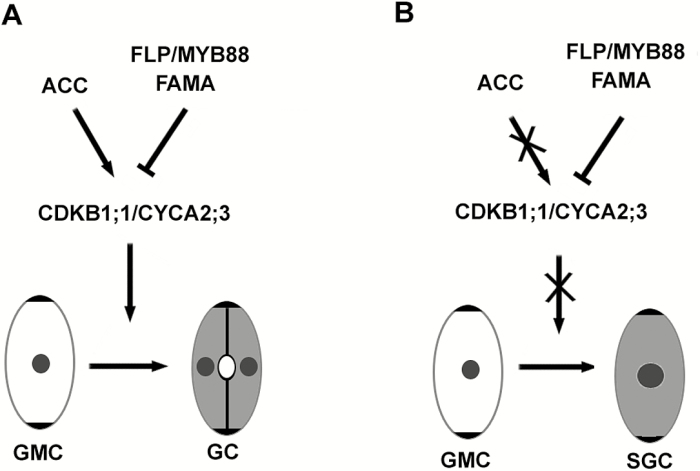
Model of the function of aminocyclopropane-1-carboxylic acid (ACC) in the symmetric division of guard mother cells (GMCs). (A) Under normal conditions, ACC and FAMA/FLP/MYB88 maintain the appropriate expression of *CDKB1;1* and *CYCA2;3* in GMCs and ensure their symmetric division into GCs. (B) When ACC synthesis is blocked, the expression of *CDKB1;1* and *CYCA2;3* is inhibited, which disrupts the symmetric division of GMCs and then leads to the formation of single guard cells (SGCs).

Our study has shown that ACC functions as an independent signal molecule to regulate the division of GMCs in Arabidopsis. Based on our results, we present a working model for ACC-mediated GMC division (Fig. 8). In this model, ACC is crucial for the symmetric division of GMCs into GCs. ACC positively regulates and FAMA/FLP/MYB88 negatively regulates the expression of *CDKB1;1* and *CYCA2;3*, which are responsible for the division of GMCs. Under normal conditions (Fig. 8A), ACC and FAMA/FLP/MYB88 maintain the appropriate expression of *CDKB1;1* and *CYCA2;3* in the GMCs and ensure their symmetric division into GCs. When ACC synthesis is inhibited (Fig. 8B), the expression of *CDKB1;1* and *CYCA2;3* is down-regulated, which disrupts the symmetric division of GMCs and then leads to the formation of SGCs.

A recent report has demonstrated that the bHLH protein MUTE directly binds to the promoters of *CDKB1;1* and *CYCA*s and up-regulates their gene expression (Han *et al.*, 2018*a*). Here, we found that ACC is a positive regulator of *CDKB1;1* and *CYCA*s. It is unclear whether MUTE functions downstream of ACC. Further investigation of the signal components responsible for ACC-mediated GMC division will provide new insights into the mechanisms underlying the terminal division of GMCs.

## Supplementary data

Supplementary data are available at *JXB* online.

Fig. S1. AVG treatment results in SGC formation in the hypocotyl epidermis of wild-type Arabidopsis.

Fig. S2. SGCs in the *acs* octuple-mutant.

Fig. S3. ACC and ethephon treatment fail to induce SGCs in the hypocotyl epidermis of the wild-type.

Fig. S4. No extra division of GMCs is observed in the hypocotyl epidermis of etiolated *eto1-1* seedlings.

Fig. S5. Ethephon, AIB and Co^2+^ have no effect on the division of GMCs in the *fama-1* and *flp-1myb88* mutants.

Fig. S6. There are no SGCs in the *acs* double-, quadruple-, pentuple-, hexuple-, and heptuple-mutants.

Fig. S7. Ethylene signaling has no effect on the expression of *CDKB1;1* and *CYCA2;3*.

Table S1. Primers used for qRT-PCR in this study.

Supplementary Figures S1-S7 and Table s1

## Author Contributions

JY, XZ, and XC designed the experiments; XZ, JY, and YW performed the experiments; XZ, JY, GZ, and XC analysed the data; XZ, GZ, GL, and XC wrote the manuscript; all the authors read and approved the final manuscript.
