# Supplementary material for: Aminocyclopropane-1-carboxylic acid is a key regulator of guard mother cell terminal division in Arabidopsis thaliana
Source: J Exp Bot. 2018 Nov 21;70(3):897–908. doi: 10.1093/jxb/ery413 (PMC6363092; doi:10.1093/jxb/ery413)
Supplement: Supplementary Figures S1-S7 and Table s1 [file ery413_suppl_supplementary-figures-s1-s7_table-s1.pdf]

Aminocyclopropane-1-Aarboxylic Acid (ACC) is a key regulator of Guard Mother Cell (GMC) Terminal Division in *Arabidopsis thaliana*

Jiao Yin[1†], Xiaoqian Zhang[1†], Gensong Zhang[1†], Yuanyuan Wen[1], Gang Liang[2*], Xiaolan Chen[1*]

[1]School of Life Sciences, Yunnan University, Kunming, Yunnan 650091, China.
[2]CAS Key Laboratory of Tropical Plant Resources and Sustainable Use, Xishuangbanna Tropical Botanical Garden, Chinese Academy of Sciences, Kunming, Yunnan 650223, China

[†] These authors contributed equally to this work.

***Correspondence:**

Xiaolan Chen

chenxiaolan@ynu.edu.cn

Gang Liang

lianggang@xtbg.ac.cn

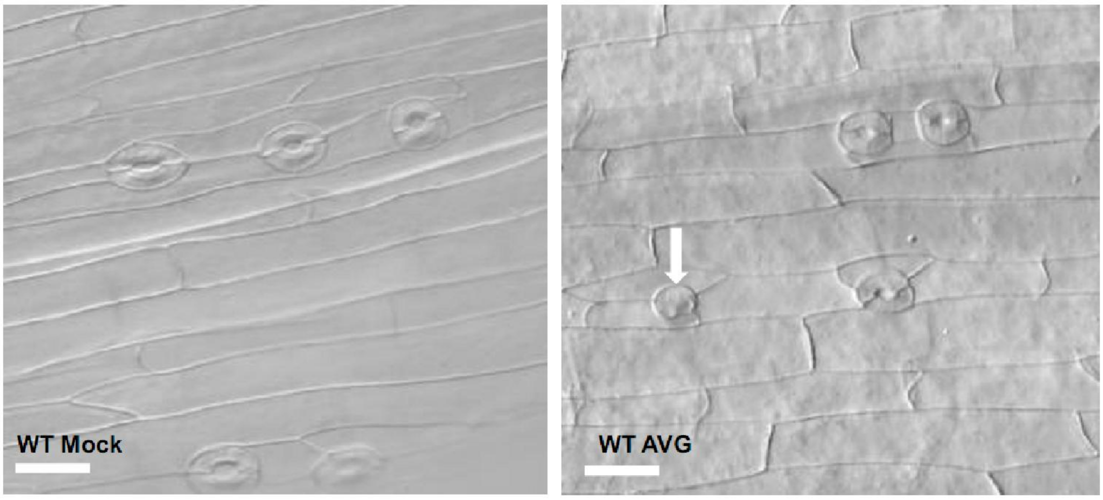

Supplementary Figure 1. AVG treatment results in SGC formation in hypocotyl epidermis in wild type Arabidopsis.

SGCs in the epidermis of hypocotyl. Arrow indicates SGC. Bars=20μm.

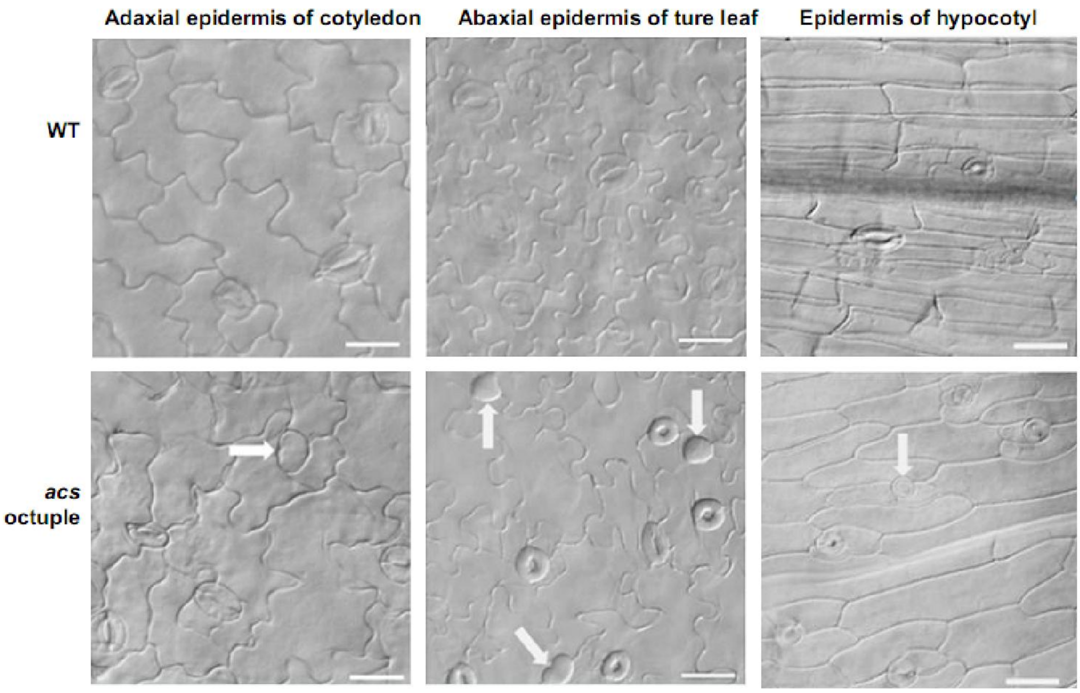

Supplementary Figure 2. SGCs in the *acs* octuple mutant.

SGCs on the adaxial epidermis of cotyledon, abaxial epidermis of true leaf and epidermis of hypocotyl in *acs* octuple mutant. White arrows indicate SGCs. Bars=20μm.

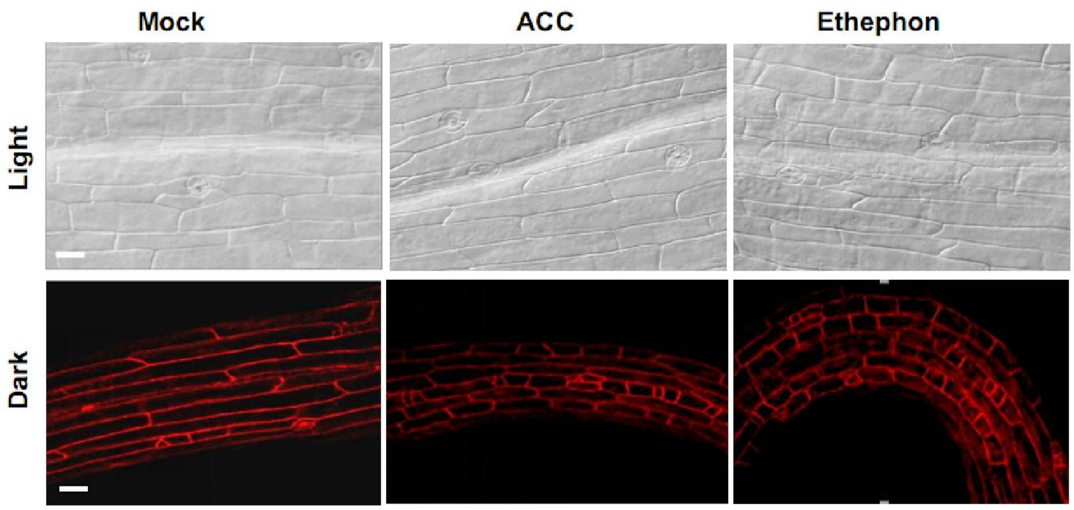

Supplementary Figure 3. ACC and ethephon treatment fail to induce SGCs in the hypocotyl epidermis of Wild type.

Hypocotyl epidermis. Wild type seedlings grown on media with ACC or ethephon under light or dark were used for stomatal analysis. Bars=20μm.

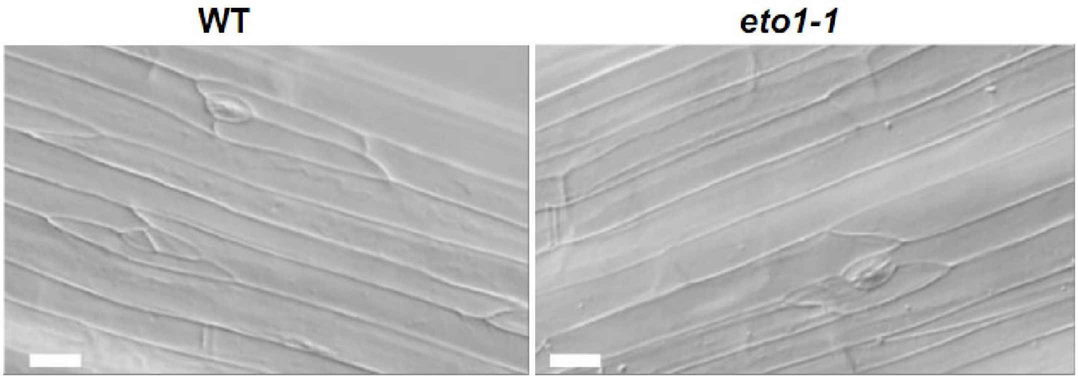

Supplementary Figure 4. No extra division of GMCs on the hypocotyl epidermis of etiolated *eto1-1* seedlings.

Hypocotyl epidermis. Wild type and *eto1-1* mutants were grown under dark. Bars=20μm.

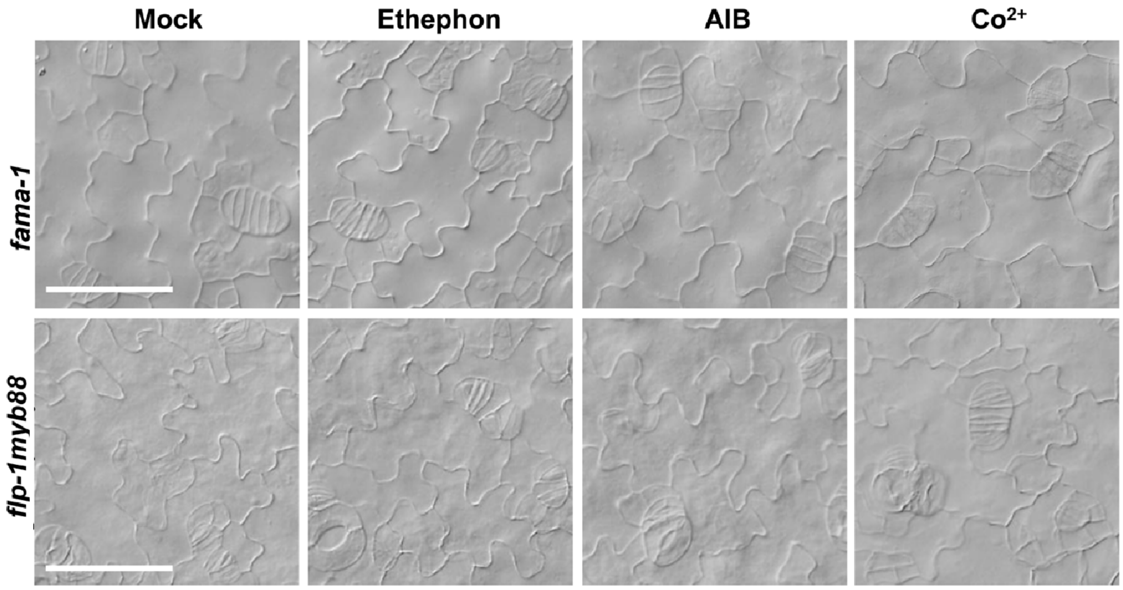

Supplementary Figure 5. Ethephon, AIB and Co^{2+} have no effect on the division of GMCs in *fama-1* and *flp-1myb88* mutants.

Abaxial epidermis of cotyledon in the *fama-1* and *flp-1myb88* mutants. Bars=20μm.

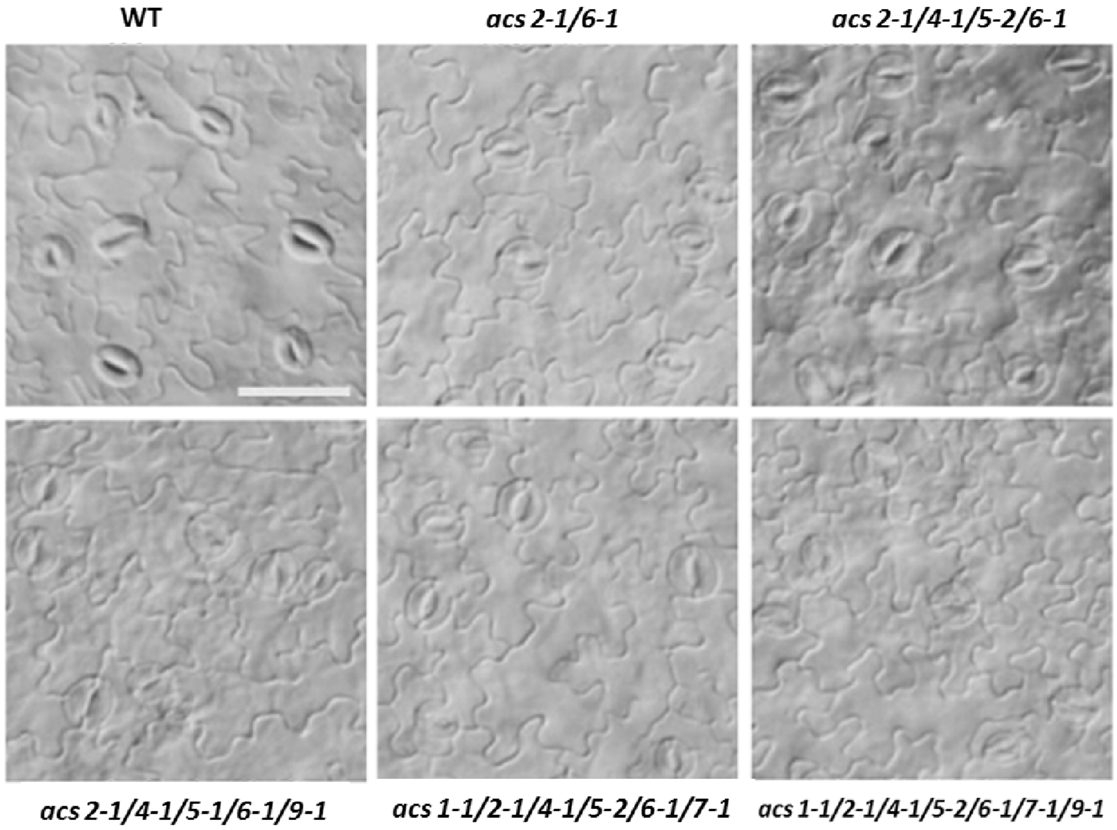

Supplementary Figure 6. No SGC in the *acs* double, quadruple, pentuple, hexuple and heptuple mutants.

Abaxial epidermis of cotyledon. Bars=20μm.

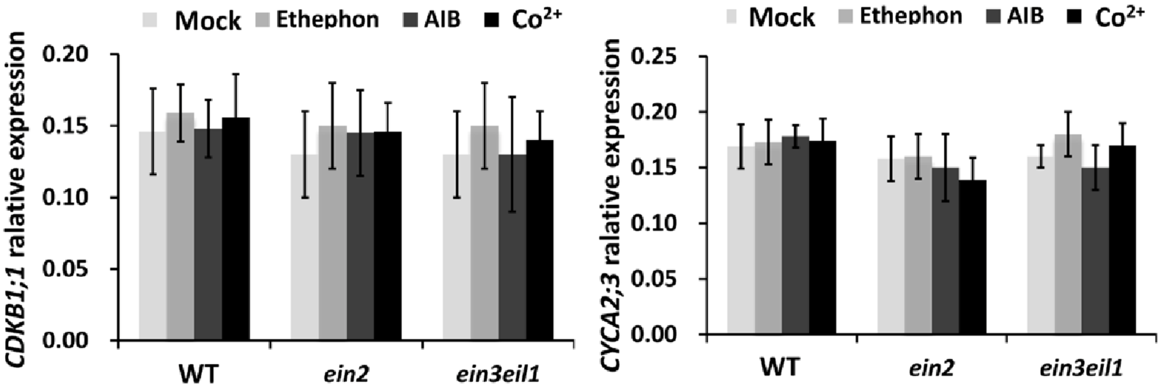

Supplementary Figure 7. Ethylene signaling has no effect on the expression of *CDKB1;1* and *CYCA2;3.*

Relative mRNA level of *CDKB1;1* and *CYCA2;3* relative to *KAT1*. Error bars correspond to the SD on three biological triplicates. The values marked by one asterisk are significantly different from the corresponding value with Mock (Student's *t* test; P<0.01; n= 3).

Supplementary Table 1. Primers used for qRT-PCR in this study.

Primer name	Sequence
CDKB1;1-F	5'-AGTCCAGAAACCAACACAG-3'
CDKB1;1-R	5'-CAACAAGCTTACCAGTTCC-3'
CYCA2;3-F	5'-GTTCCCTTGCCTCTGCTTTGC-3'
CYCA2;3-R	5'-GCTCGCTTCTTCCTCTTGTTGG-3'
KAT1-F	5'-ACCGCTGGAAGAAGTTACGCTAACG-3'
KAT1-R	5'-AGCGTTGTCCGCATTGGTGATCTTT-3'
